# Multiple center listing for organ transplantation in the United States: time to reform?

**DOI:** 10.3389/frtra.2025.1677463

**Published:** 2025-10-01

**Authors:** Emmanouil Giorgakis, Keren Ladin, Sher-Lu Pai, Dimitrios Moris, Esteban Calderon, Oya Andacoglu, Nazia Selzner, Paulo N. Martins

**Affiliations:** ^1^ Department of Surgery, Division of Transplant, UNC Chapel Hill, Chapel Hill, NC, United States; ^2^ Department of Community Health, Tufts University, Boston, MA, United States; ^3^ Department of Anesthesiology and Perioperative Medicine, Mayo Clinic, Jacksonville, FL, United States; ^4^ Transplant Surgery, Medstar Georgetown Transplant Institute, Washington, DC, United States; ^5^ Division of Transplant Surgery, Presbyterian/St. Luke’s Medical Center, Denver, CO, United States; ^6^ Ajmera Transplant Center, University of Toronto, Toronto, ON, Canada; ^7^ Department of Surgery, Oklahoma Transplant Institute, Oklahoma, OK, United States

**Keywords:** equity, ethics, multiple listing, organ transplantation, transplant access

## Introduction

Multiple listing (ML) allows transplant candidates to be registered at more than one transplant center. The United States (US) is the only country known to allow this practice (Table 1). In Europe, ML is strictly forbidden (1). Although ML may support individual **autonomy** and choice—values deeply embedded in the American culture—it raises ethical dilemmas. Patients with greater financial means and mobility may be more capable of securing places on multiple transplant waitlists, allowing socioeconomic status to impact the probability of transplant. This opinion paper explores ML through the lens of the ethical principles **of autonomy**, **equity**, **justice**, and **utility** used in transplantation (2).

**Table 1:** Global overview of multiple listing policies in deceased donor organ transplantation.

Country/Region	Multiple Listing (ML) Policy	Policy Description	Rationale
United States	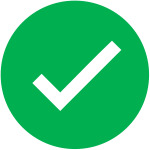	Patients may register at multiple transplant centers across different regions.	Promotes patient autonomy and choice; It may favor those with greater resources and mobility.
Argentina	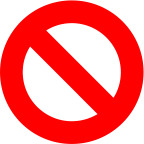	National Institute for Organ Procurement and Transplantation oversees allocation.	Focuses on fairness and reducing inequalities in organ distribution.
Australia	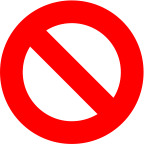	Centralized national allocation through the Organ and Tissue Authority.	Ensures access based on medical need without geographic bias.
Brazil	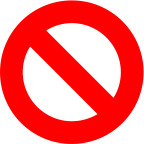	National Transplant System coordinates organ allocation; single listing enforced.	Aims to provide equitable access and reduce regional disparities.
Canada	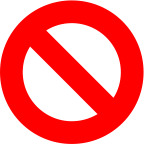	Centralized system with regionally coordinated allocation; patients listed at one center.	Aims for fairness and transparency in organ allocation.
Chile	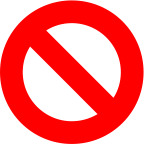	National Transplant Coordination Unit manages a centralized waitlist.	Ensures equitable access and efficient organ utilization.
China	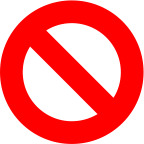	China Organ Transplant Response System coordinates national allocation.	Focuses on fairness and reducing regional disparities.
Colombia	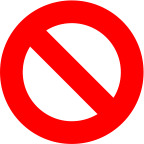	National Network for Organ Donation and Transplantation coordinates allocation.	Aims for fairness and transparency in organ distribution.
Eurotransplant (Austria, Belgium, Croatia, Germany, Hungary, Luxembourg, Netherlands, Slovenia)	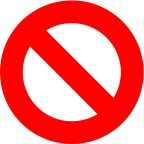	Centralized system for multiple European countries.	Balances organ supply and demand across borders, ensuring equitable allocation.
France	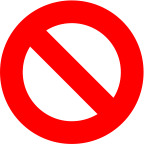	Managed by Agence de la Biomédecine with a national waiting list.	Focuses on equitable access without financial or geographic bias.
Germany	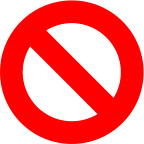	Part of Eurotransplant; centralized allocation across member countries.	Supports fairness and equity across participating nations.
Greece	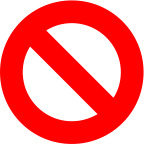	Centralized allocation managed by the National Transplant Organization (NTO), ensuring that patients are listed at a single transplant center.	Maintains equity in organ allocation
India	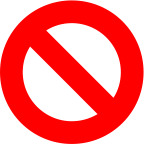	National Organ and Tissue Transplant Organization manages centralized allocation.	Ensures equitable access and transparency in organ distribution.
Iran	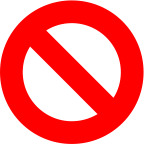	Ministry of Health oversees a centralized allocation system.	Aims to provide fair and efficient organ distribution.
Israel	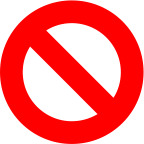	National Transplant Center manages a centralized waitlist.	Ensures equitable access and prioritizes medical urgency.
Italy	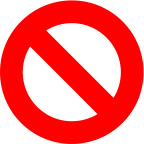	Centralized allocation managed by the National Transplant Center.	Ensures organ allocation based on medical urgency and need.
Japan	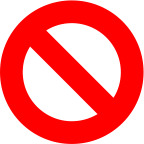	Japan Organ Transplant Network oversees a centralized allocation system.	Ensures fairness and transparency in organ distribution.
Mexico	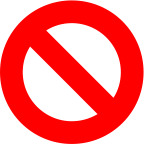	National Transplant Center manages a centralized waitlist system.	Prioritizes equitable access and reduces regional disparities.
New Zealand	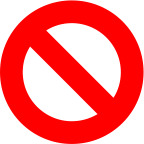	Organ Donation New Zealand manages a centralized waitlist.	Ensures fairness and transparency in organ allocation.
Saudi Arabia	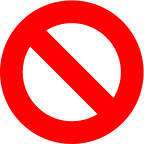	Saudi Center for Organ Transplantation oversees centralized allocation.	Focuses on fairness and reducing regional disparities.
Scandinavia (Scandiatransplant)	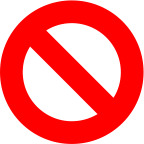	Shared waiting list among Nordic countries with centralized coordination.	Promotes equity and fair access across member nations.
South Africa	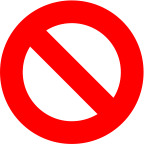	South African Transplant Society coordinates organ allocation.	Aims to provide equitable access and efficient distribution.
South Korea	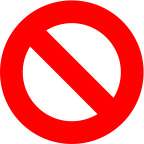	Korean Network for Organ Sharing manages a centralized waitlist.	Aims to provide equitable access and efficient organ allocation.
Spain	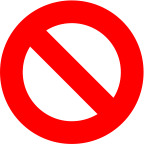	National Transplant Organization oversees allocation with a centralized system.	Prioritizes equitable access and efficient distribution.
United Kingdom	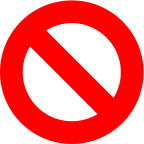	Centralized allocation managed by NHS Blood and Transplant; single national waitlist.	Ensures equitable access based on medical need, minimizing disparities.

## Background

**Organ Procurement and Transplant Network (OPTN)** requires transplant centers to notify candidates that ML is allowed (2–5). Transplant centers may decide whether to accept a patient who has already been listed elsewhere (3). The **Final Rule**, a US National Policy that dictates the protocol for all deceased organ donations, states that “organs should be allocated across the widest possible geographic area, while accounting for the urgency of each recipient's need” (5). **The American Medical Association Code of Medical Ethics** asserts that “organs should be treated as a national resource without geographical limitations unless the transportation of the organ would compromise its viability for transplantation” (6).

ML has an extensive history in the OPTN (3). In August 1987, the OPTN Board of Directors passed a resolution to allow patients to ML. The policy almost instantly faced criticism primarily due to concerns over potential inequities from these multiple registrations. These concerns led to OPTN public comment invite, Ethics and OPTN Board of Director Meetings, which ultimately allowed for the 1987 resolution to persist. ML policy was brought to the forefront again in 1994 and in 2001, with similar concerns. In November 2003, the OPTN Board of Directors voted to not restrict the policy and approved amendments regarding better patient education on their right to pursue ML.

Despite the 2003 decision, the ongoing controversy led to OPTN Ethics Committee analysis in early 2022, which delivered a White Paper on the Ethical Evaluation of ML (3). The Committee specifically distinguished between pursuing multiple evaluations and ML. It concluded that “retaining the existing ML policy does promote equitable access to transplantation”. “Widespread availability of ML may undermine equity and utility” (…) “However encouraging ML for patients who are disproportionately difficult to match is ethically justifiable to promote their equal access to transplant” (3).

Access to healthcare and organ transplantation vary significantly across the US (4–7). ML was introduced to combat geographic disparities through enabling patients to circumvent regional differences in organ availability and waitlist dynamics, which persist despite national allocation reforms such as the acuity circles policy (3, 7, 8). However, ML may be “good for a few, but no solution for the organ shortage” (9). There have been concerns over ML shifting inequities rather that providing a solution (9). Patients with private insurance and higher education are more likely to be listed and transplanted sooner compared to those with Medicaid or no insurance (10). A United Network for Organ Sharing (UNOS) analysis by Merion et al. on 81,481 kidney and 26,260 liver transplant candidates (1995–2000), identified approximately 5.8% of kidney transplant and 3.3% of LT candidates as multiple-listed (11). Non-white race, older age, non-private insurance, and lower educational level were associated with lower ML rates (11). Transplant rates were higher for ML candidates (11). Another UNOS study on 59,557 liver transplant candidates (2005–2011) showed 2.3% being multi-listed (8). Among these patients, 67.6% underwent transplant at the secondary listing Donation Service Areas. ML recipients had shorter wait-times, lower median Model for End-Stage Liver Disease (MELD) scores, were more likely to be white, college educated, and with private insurance (9). Other studies have shown that ML considerably improves transplant rates and survival on low MELD patients (7). A more recent analysis reports that ML outside the primary OPTN/UNOS region was more likely on patients with college-level of education (33% vs. 21%; *P* < .001) and significantly higher median annual income by ZIP code (12).

UNOS/OPTN studies on lung and heart transplant populations reached similar conclusions. On 43,578 patients waitlisted for lung transplant during 2006–2014, 2.3% had multiple registrations (13). ML was associated with increased likelihood of transplant (aHR 2.74% CI 2.37–3.16), but no change on waitlist mortality. Younger age, female gender, white race, antibody sensitization, higher education, and cystic fibrosis were independently associated with ML (13). Another UNOS study on lung transplant candidates (2005–2018) using Social Deprivation Index (DPI) to assess disparities between ML and single listing (SL), concluded that ML patients were more likely to be transplanted if they had been multiple-listed early (14). On the same study, ML candidates had lower median DPI, were more likely to be females, and had lower median Lung Allocation Score (LAS) (14). A contemporaneous UNOS study on lung transplant patients showed that ML was associated with substantial increase in the probability of transplant, with no impact on survival (15). Notably, ML patients had lower LAS (i.e., were less sick) compared to the single-listed (1, 15). The ML group patients were more likely to be white, female, suffering from cystic fibrosis, with higher level of education, and private insurance (15). A recent UNOS study encompassing lung and heart transplant recipients showed that ML candidates were more likely to be privately insured (58.9% vs. 51.1%), less likely to be Medicaid (5.8% vs. 10.3%), and living in ZIP codes with higher median incomes (all *P* values < .00001) (16). These multiple-listed patients had higher transplant rates despite longer wait times, and lower waitlist mortality (16).

Patients are often multiple-listed in programs within the same metropolitan region, therefore competing for the same donor source as their primary enlisting location perhaps reflecting variations in program organ acceptance practices rather than donor availability. Current ML prevalence may be much higher than previously reported in urban areas with high transplant center density (anecdotal data).

From a public opinion standpoint the perception is that ML is unfair, as exemplified by the publicized case of Steve Jobs who had “cut the line” and got transplanted through ML and on OPTN public comment reports on the policy (3, 17).

## Discussion

The above observations question whether ML in its current form remains an ethically and socially acceptable strategy (1, 9, 18).

The rest of our discussion will be rooted in the principles recognized in transplant ethics. These principles are considered the foundation of an ethical transplant system (2, 3).

**Autonomy** (2, 18–20). Patients should have the right to self-determination in their medical decisions, provided these choices do not unduly harm others. However, deceased donor organ transplantation is a zero-sum game. If ML increases the chances for one patient to access deceased donor organs, it inevitably decreases the relative chance for others to access the same finite organ supply.

**Equity** (2, 18). In healthcare, equity means allowing people to be treated differently if this is necessary to achieve equal health outcome, especially across populations with systemic disadvantages. ML may promote equity for certain “hard-to-match” patient populations that are comparatively harder to be transplanted. In the context of solid organ transplant allocation, “hard-to-match” patients are those who face significant barriers to receiving a suitable organ due to factors such as uncommon blood type or small size, highly sensitized patients due to prior transplants, or unique medical urgency that is not fully captured by standard allocation scores. In liver transplantation, this term most commonly refers to candidates with anticipated challenging anatomy (e.g., polycystic liver patients, retransplant candidates, or patients requiring complex vascular reconstruction or combined transplants), pediatric patients or small sized adults, or those with conditions for which standard scoring systems underestimate urgency or disease severity—such as significant ascites or pruritus not reflected on the MELD score, hepatocellular carcinoma or other hepatic malignancies that would benefit from transplant but remain beyond the current MELD exception criteria but may still benefit from an “extended criteria” graft not available locally, etc. (20, 21).

Those “hard-to-match” or “worst-off” patients may face inherent disadvantages within a SL allocation system. ML may be necessary to “level the playing field” and achieve a likelihood of transplantation comparable to less complex patients, should they wish to pursue.

In his book A Theory of Justice, John Rawls distinguishes “formal equality of opportunity” as merely removing legal barriers so all can compete for the same good, from “fair equality of opportunity”, which poses that individuals with similar abilities have equal life chances regardless of their socioeconomic background (22). Fair equality of opportunity requires active measures to compensate for uneven starting points, such as access to quality education and resources (8). Based on Rawls, formal equality of opportunity determines that ML should be available to all without discriminations, while fair equality demands that individuals with similar needs and willingness to pursue ML should have genuinely similar chances of success (22). Multiple previous studies have shown that, even though formal equality of opportunity exists (ML is permissible to all candidates), fair equality is yet to be reached (1, 9, 11–16).

**Distributive Justice** (2, 18, 23–25). Rawls' conception of distributive justice requires guaranteeing *equal basic liberties for all*, while allowing social and economic inequalities only if they both enhance the situation of the least advantaged (*the difference principle*) and preserve genuine equality of opportunity *(fair equality of opportunity)* (22)*.* Highly sensitized kidney transplant candidates, transplant oncology patients who are not eligible for exception points, and pediatric recipients may all be evaluated or listed locally, but they can face major disadvantages if restricted to a single transplant center. For example, a local program may not use extended criteria grafts (such as older or DCD livers) for patients with decompensated cirrhosis who have low MELD scores, or for cancer patients who do not qualify for MELD exception points. Similarly, the local kidney program may lack desensitization protocols for highly sensitized patients, or the local liver team may be more conservative in transplanting pediatric patients, or those needing retransplantation, complex implantation approach, graft splitting or reduction or those eligible for living donation (and available donors), thus delaying their transplant. For these “worst-off” patients, ML can significantly improve access to transplant by allowing them to seek care at centers that provide these additional options. Limiting their ability to pursue ML risks causing harm by cutting off a viable path to life-saving treatment. Supporting ML for medically complex or disadvantaged patients therefore aligns closely with the principle of distributive justice.

A counterpoint to ML is that it may allow one patient to receive an organ that could otherwise have gone to a candidate limited to their primary listing center, thereby constraining the latter's autonomy. For example, an older DCD liver might be allocated to a multiple-listed “imported” patient instead of a local low-MELD candidate who was hoping for extended criteria grafts to become available. If ML reliably accelerated transplants for higher-acuity patients, such prioritization could be justified. However, UNOS data does not support this—ML does not consistently benefit the sickest patients (15, 26). Therefore, from a distributive justice perspective, the benefits of ML are nuanced: while it can help certain patients, it does not consistently advantage those with the most urgent need.

**Procedural Justice** (2, 19, 22, 27). Procedural justice means that an outcome is considered fair when it is reached through a fair process. In transplantation, this does not mean guaranteeing the same outcome for every candidate but ensuring that evaluation and decision-making are conducted in an unbiased, equitable, and consistently applied manner. With regards to ML, transplant programs are required to inform patients of their right to pursue it should they wish to do so. However, the degree to which the programs convey and how well patients understand this information remains unclear. Plus, the willingness of a program to evaluate a patient that has been evaluated elsewhere may vary. Moreover, while patients are theoretically free to pursue ML to find the best fit, programs and insurance providers, exercising their own autonomy, can refuse to accept. Patients may spend a significant amount of time and resources only to be rejected. Insurance providers can restrict patient choice by limiting coverage to specific institutions (e.g., Centers of Excellence) (28). Uninsured patients are often being entirely excluded from accessing transplantation.

**Utility** (2). In transplant ethics, utility refers to the principle of maximizing the overall benefit produced by scarce donor organs. This means prioritizing transplants where the organ is most likely to provide the longest graft and patient survival, improve quality of life the most; and use resources most effectively. That said, utility must be balanced against other ethical principles in transplantation such as justice (fair access) and respect for persons (honoring patient autonomy) (2). ML may increase organ utilization for allografts that otherwise may have been discarded. For example, livers not suitable for very sick candidates may still be beneficial for transplant oncology or low MELD (<15) patients. ML has increased transplant rates and survival such populations (9). Also, Centers of Excellence may offer treatments not available locally and higher quality-based care, resulting in more transplants, hence higher graft utilization, and better outcomes. Finally, competition between transplant centers intensifies performance.

On the other hand, managing care across multiple distant programs can lead to fragmented information, duplicate testing and increased cost for patients, programs and insurance payers, thus compromising utility. ML may unnecessarily burden the transplant apparatus, congesting the transplant evaluation referrals, driving up operational costs for all. As insurance companies vary on their coverage for ML evaluations, candidates may need to cover all or part of pre-transplant costs for each additional listing center in addition to the costs for travel to different centers. Finally, while ML could reduce wait times and increase overall transplant numbers and graft utilization (e.g., by utilizing organs that would otherwise have been discarded due to lack of appropriate recipient), available OPTN data does not clearly support this (26). This may be partially explained by the fact that many multiple-listed patients are hard-to-match cases who inherently face longer waits.

## Conclusions

SL has been common practice outside the US. It may guard transplant equity and justice irrespective of the patient's location, demographics or status. SL would unclutter the waitlists, reducing resource waste on waitlist maintenance and multiple workups and is better aligned with the societal sentiment over better-resourced waitlisted patients “cutting the line”. ML, on the other hand, may increase the chances of successful transplantation in “worst-off” cases and potentially enhance organ utilization. Yet, it still presents an ethical challenge. It is, therefore, crucial to define the occasions where ML is justified and limit its application on such cases—thus optimizing beneficence while minimizing distribution injustice.

Our analysis points to the fact that autonomy is commonly understood as a principle that, while critical, does not supersede others, and can be understood as allowing patients to act in a way consistent with their preferences so long as that behavior does not negatively interfere with the autonomy or wellbeing of others.

While the transplant community cannot rectify all societal disparities, it has a moral obligation to address policies that exacerbate preventable inequalities within transplantation itself. More research, opinion polls, funding and policies towards increasing donation and organ utilization, may discover ways to further improve the system.
